# A New Look at Immunogenetics of Pregnancy: Maternal Major Histocompatibility Complex Class I Educates Uterine Natural Killer Cells

**DOI:** 10.3390/ijms25168869

**Published:** 2024-08-15

**Authors:** Manon Bos, Francesco Colucci

**Affiliations:** 1 Department of Pathology, Leiden University Medical Center, 2333 ZA Leiden, The Netherlands; 2 Department of Obstetrics and Gynaecology, University of Cambridge School of Clinical Medicine, NIHR Cambridge Biomedical Research Centre, Addenbrooke’s Hospital, Cambridge CB2 0QQ, UK; 3 Centre for Trophoblast Research, University of Cambridge, Cambridge CB2 1TN, UK

**Keywords:** maternal-fetal interface, uterine natural killer cell, NK cell education

## Abstract

Our incomplete knowledge of maternal–fetal interface (MFI) physiology impedes a better understanding of the pathological mechanisms leading to pregnancy complications, such as pre-eclampsia and fetal growth restriction. At the MFI, uterine natural killer (uNK) cells do not attack fetal cells but engage in crosstalk with both fetal and maternal cells to support feto-placental development. However, mother and fetus are genetically half-mismatched and certain combinations of variable immune genes—human leukocyte antigens (HLAs) and killer-cell immunoglobulin-like receptor (KIR), indeed, the most variable gene sets in the genome—associate with pregnancy outcomes, suggesting that these interactions regulate uNK cell function. How do these interactions influence the physiology and pathology at the MFI? Uterine NK cell function is regulated by both maternal and fetal Major Histocompatibility Complex (MHC); however, evidence for fetal cells educating uNK cells is lacking, and new evidence shows that maternal rather than fetal MHC class I molecules educate uNK cells. Furthermore, uNK cell education works through self-recognition by the ancient and conserved NKG2A receptor. Pregnant mice lacking this receptor produce normal litter sizes, but a significant portion of the offspring have low birthweight and abnormal brain development. Evidence from a genome-wide association study of over 150,000 human pregnancies validates the finding because women whose NKG2A receptor is genetically determined to engage their own MHC class I molecules are exposed to lower risk of developing pre-eclampsia, suggesting that maternal uNK cell education is a pre-requisite for a healthy pregnancy and, likely, for healthy offspring too.

## 1. Introduction

From an immunological point of view, pregnancy is enigmatic because it involves close contact of cells from two genetically different individuals. Hence, a dominant question for several decades has been, “Why the mother’s immune system does not reject the fetus?” [1]. We now know that, in fact, maternal immune cells in the uterus do not attack fetal cells but instead engage in a crosstalk. Nevertheless, the maternal immune system does recognize fetal antigens and responds to them; allo-antibodies can be formed against fetal HLA antigens, fetal erythrocytes or platelets, and these associate with serious fetal complications, such as hemolytic disease of the fetus [2].

The healthy interactions between maternal immune cells and fetal trophoblast cells most likely facilitate pregnancy rather than impede the development of the semi-allogenic fetus. Indeed, many immune cell types are found at the maternal–fetal interface (MFI), and different mechanisms have evolved to regulate their function. For instance, the syncytiotrophoblast completely lacks the expression of any type of Human Leukocyte Antigen (HLA) molecule. Nevertheless, extravillous trophoblast cells do express polymorphic HLA-C antigens and could be recognized by HLA-C-restricted alloreactive maternal T cells and by immune maternal uterine natural killer (uNK) cells. The interactions between HLA-C and Killer-cell Immunoglobulin-like Receptors (KIRs) are thought to be essential for normal pregnancy [3]. Genetic epidemiology studies also show that certain combinations of fetal HLA-C and maternal KIR genetic variants that potentially suppress uNK cell function are associated more frequently with pre-eclampsia and low birth weight [4].

In this article, we review the general features of NK cell function and education and the unique interactions at the MFI between uNK cells and trophoblast. We attempt to provide general background knowledge on the immunology at the MFI, discuss different views based on knowledge acquired in transplantation and tumor immunology, and argue that interactions between inhibitory receptors on uNK cells with maternal HLA rather than fetal HLA influence pregnancy outcome through the process of uNK cell education. Also, the HLA-B–HLA-E–NKG2A pathway is discussed as a new mechanism for uNK cell regulation [5].

## 2. Results

### 2.1. NK Cell Function, Diversity, and Education

We focused on uterine NK cells and their interaction with fetal trophoblasts at the MFI in this review. However, NK cells are found in most tissues and in blood. Blood NK cells are the most studied. In the 1970s, an immune cell population was discovered in the mouse spleen and human peripheral blood with spontaneous cytotoxicity, setting them apart from other cytotoxic cells [6,7]. These cells were named natural killer cells due to their ability to kill target cells without the need for prior stimulation [8]. A decade later, the missing-self hypothesis was formed based on the spontaneous cytotoxicity of NK cells to cells that do not express Major Histocompatibility Complex (MHC) class I molecules [9]. This hypothesis was counterintuitive in an era when the recently discovered MHC restriction for T-cell specificity was so influential on immunologists. However, the discovery of inhibitory receptors for MHC class I on NK cells gave a molecular framework to the hypothesis. First, it was discovered that mouse NK cells expressing the inhibitory Ly49A receptor were tolerant of tumor cells expressing cognate MHC class I molecules (H-2 in the mouse), while Ly49A-negative NK cells effectively lysed H-2 positive tumor cells because they could not recognize self-MHC [10]. Then, human KIR, with similar functions, were identified [11]. While the first Ly49 and KIR receptors discovered were inhibitory, it soon emerged that there are also activating variants of Ly49 and KIR [12]. These variable receptors, expressed in a variegated fashion on individual NK cells and, in combination with other cell surface markers, generate heterogeneous repertoires of NK cells. Ly49 and KIR are acquired late in NK cell development. The inhibitory receptor CD94/NKG2A (hereafter referred to as NKG2A), which was discovered later than Ly49 and KIR, recognizes the non-classical HLA-E in humans and Qa-1 in mice [13,14]. NKG2A also educates NK cells in both species and is expressed earlier in NK cell development than Ly49 in mice and KIR in humans. The developmental stages of NK cells are defined by several surface receptors, have been extensively reviewed, and are beyond the scope of this review [15].

In summary, without prior sensitization or antigen-specificity, NK cells detect and respond to cells under stress, such as infected cells or tumor cells. NK cell education (also known as NK cell licensing) finetunes their functional competence [16]. Inhibitory receptors for MHC class I molecules educate NK cells to be tolerant of self and license them to functional competence [12,17]. NK cells that lack inhibitory receptors for self-MHC class I molecules are hyporesponsive and have a low potential to attack normal cells [18].

### 2.2. Uterine NK Cells

**Figure 1:**
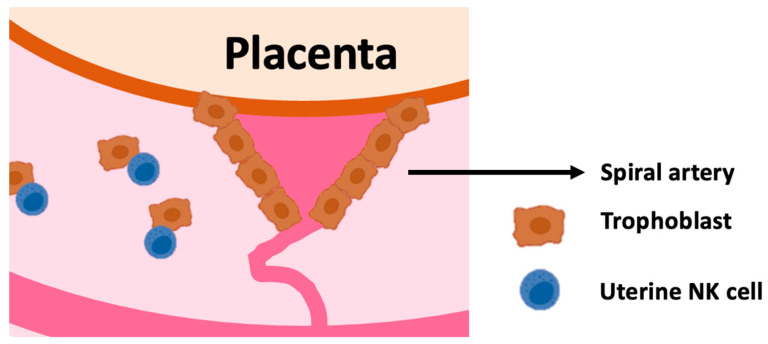
Uterine NK cells interact with extravillous trophoblasts: Extra villous trophoblasts invade in the spiral arteries, decidua and myometrium of the uterus. Uterine NK cells engage in crosstalk with trophoblast. Trophoblasts are a unique kind of extraembryonic cells that pose interesting immunological challenges [1].

We have discussed elsewhere [19] why uterine NK cells are referred to as uNK or dNK cells. Uterine NK cells are found in the non-pregnant uterus, in the decidua, and, in mice, also in between the two muscle layers of the uterine wall (Figure 1). Decidual Natural Killer (dNK) cells are exclusively found in the decidua. In humans, uNK and dNK cells are essentially synonymous because NK cells are only found in the decidua and not in the muscular layer of the uterine wall.

In the endometrium, approximately 45% of CD45-positive cells are NK cells, reaching 70% during the first trimester in the decidua. As gestation progresses, this number drops to 15–20% [20]. For a successful pregnancy, appropriate trophoblast invasion and the correct formation of the MFI are essential, and uNK cells regulate both processes [21,22]. Uterine NK cells appear to control the invasion of trophoblasts into the maternal endometrium and the formation of spiral arteries. Incomplete spiral artery transformation is associated with pre-eclampsia and fetal growth restriction, while excessively deep trophoblast invasion results in placenta accreta. Hence, uNK cells are key players in the MFI in health and disease [23].

The origin of uNK cells is still debated and there is evidence for their in situ origin, as well as for their origin from circulating precursor cells. In receivers of a transplanted uterus, uNK cells are host-derived, suggesting an origin extrinsic to the uterus [24]. However, a parabiosis study in mice shows that a significant proportion of uNK cells derive from tissue resident cells [25].

The phenotype of uNK cells is distinct from that of blood NK cells, and, like other tissue-resident NK cells, they are poorly cytotoxic. Uterine NK cells in humans are defined by the following surface marker expression: CD56^bright^CD16^−^CD9^+^CD49a^+^. In contrast, mature peripheral blood NK cells are CD9^−^ and CD49a^−^, and the majority are CD56^dim^ CD16^+^ [20,26]. Based on single-cell RNA sequencing, three subsets of uNK cells, named decidual NK cell (dNK) subset 1-3, can be identified in humans [27]. The first and largest subset (dNK1) is defined by the expression of CD39, CYP26A1, and B4GALNT1; the second subset (dNK2) is defined by ANXA1 and ITGB2 (also known as CD18); and the third subset (dNK3), is defined by CD160, KLRB1, and ITGAE (also known as CD103) and lacks the innate lymphoid cell marker CD127 [27]. Decidual NK1 also expresses KIRs and LILRB1, a receptor with a high affinity for HLA-G on extravillous trophoblast cells, making the dNK1 subset the most likely subset to interact with extravillous trophoblast [27]. The three dNK cell populations, as identified by single-cell RNA sequencing, can also be identified by cell surface maker expression in flow or mass cytometry by gating on CD45^+^CD56^+^CD3^−^CD49a^+^ cells, with the three subsets defined by differential expression of CD39 and CD103 [20]. Uterine NK cell expression of KIRs peaks in the first trimester, coinciding with peak functional responses, as measured by degranulation, suggesting that uNK cells are most active in the first trimester at the time of the implantation [20]. The majority of uNK cells express NKG2A, which is predominant in dNK2 and dNK3 and is stable throughout gestation [20]. Later functional studies showed that all three different NK cell subsets produce factors (GM-CSF, XCL1, MIP1α, and MIP1β) that are likely to modify the invasion of extravillous trophoblast cells [28]. The dNK1 cells poorly respond to classical NK cell stimuli (PMA and ionomycin and K652 cells); in contrast, when the KIR2DS4 receptor is cross-linked on dNK1 cells, they degranulate [28]. The responsiveness of dNK1 cells may be associated with increasing KIR expression, as measured by higher granzyme B levels and greater degranulation in response to cross-linking activating KIR2DS4 [28].

Thus, uNK cells have specialized functions dedicated to achieving placental development and successful reproduction. A subset of uNK cells may become more efficient with subsequent pregnancies [29]. However, an appropriate in vitro assay to quantify uNK cell function is lacking, and we still rely on assays designed to measure functions in peripheral blood NK cells. This is clearly a limitation in the field because uNK cells are distinct from blood NK cells and most likely exert different functions. New approaches and methods, including spatial single-cell multi-omics and co-culturing with trophoblast and endometrial organoids, may shed light on the function of uNK cells in their natural context.

Before we move on, we would like to call the reader’s attention to potential biases in the field. For example, our appreciation of immunology at the MFI has historically been informed by our understanding of classical immunology, including transplantation immunology. However, there are fundamental differences between cells and molecules involved in transplantation—adaptive lymphocytes—and those involved at the MFI—mostly cells of innate immunity. Moreover, our understanding of uNK cells is incomplete and sometimes inferred based on the biology of peripheral blood NK cells. For example, there are no suitable functional assays to quantify uNK cell functions in vitro, and we rely on assays developed for blood NK cells.

### 2.3. Education of uNK Cells by KIR and HLA-C

**Figure 2:**
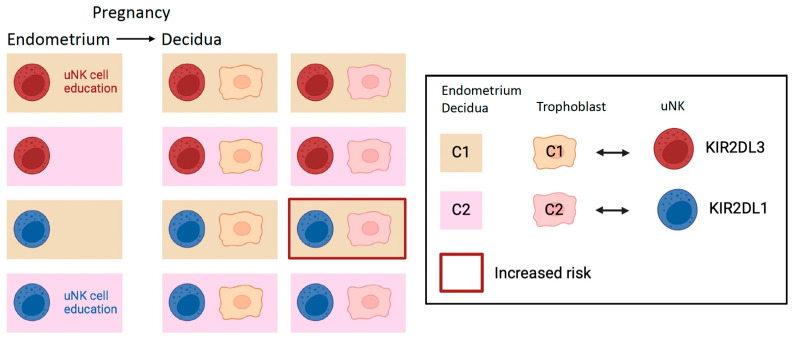
uNK cell education and inhibition by KIR and HLA-C1 or C2: uNK cell education may occur in the endometrium before pregnancy, and therefore, it is regulated by combinations of maternal KIR and maternal HLA-C variants. During pregnancy, fetal HLA-C2 on trophoblast may also regulate uNK cells by overly suppressing uNK cells that express the cognate inhibitory KIR2DL1 receptor. uNK cells expressing KIR2DL3 (red) are educated by maternal HLA-C1 (upper row) but not by maternal HLA-C2 (second row) in the endometrium. uNK cells expressing KIR2DL1 (blue) are not educated by maternal HLA-C1 (third row) and are educated by maternal HLA-C2 (bottom row). During pregnancy, the immunogenetics combinations of the two top rows do not pose risks for pregnancy complications, regardless of the trophoblast HLA-C allotype, because the inhibitory interactions are not too strong, and uNK cells maintain functionality. On the other hand, in HLA-C1/C1 women (third row), trophoblast HLA-C2 inhibit KIR2DL1-expressing uNK cells in the decidua, exposing women to greater risks of pregnancy complications because the uNK cells are overly inhibited. In HLA-C2 women, uNK cells are KIR2DL1-educated in the endometrium and, during pregnancy, can maintain functionality despite HLA-C2 expression by the trophoblast (bottom row).

**Figure 3:**
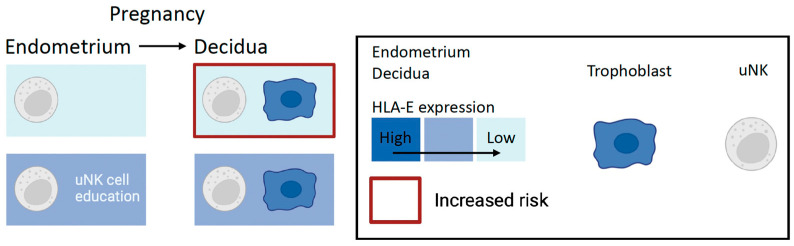
uNK cell education and inhibition by NKG2A and HLA-E. uNK cells express NKG2A. Depending on the HLA-B dimorphism in an individual, expression of HLA-E is either low (left, top row) or high (left, bottom row) in the endometrium or decidua of an individual. Genetically determined HLA-E expression levels determine whether uNK cells are educated through NKG2A in the endometrium; low HLA-E levels result in inadequate NKG2A education (left, top row) and high HLA-E levels result in adequate NKG2A education (right, bottom row). During pregnancy, trophoblast cells express the highest levels of HLA-E in the decidua and can inhibit NKG2A-expressing uNK cells. Women with inadequate NKG2A education (right, upper row) are exposed to greater risks of pregnancy complications because their uNK cells are overly inhibited. On the other hand, women with adequate NKG2A education maintain uNK cell functionality (right, bottom row).

Just like peripheral NK cells, the reactivity of uNKs may also be affected by inhibitory receptor–ligand interactions, including KIR2DL2/3 with HLA-C1, KIR2DL1 with HLA-C2, KIR3DL1 with HLA-Bw4, and NKG2A with HLA-E. These interactions educate uNK cells (Figure 2 and Figure 3). A key mechanism that trophoblast cells use to escape maternal immune recognition is a selective expression pattern of MHC class I. Villous trophoblast and the syncytiotrophoblast do not express any HLA antigen, while extravillous trophoblast cells express a specific set of HLA class I molecules: HLA-C, HLA-E, and HLA-G but not HLA-A or HLA-B [30]. Non-classical HLA-E and HLA-G vary very little, meaning that there are very few alleles in human populations, as opposed to thousands of classical HLA-A, -B, and -C alleles. HLA-C is the only polymorphic HLA expressed by the extravillous trophoblast. In principle, both maternal and fetal HLA ligands may educate uNK cells; however, evidence for fetal cells educating uNK cells is lacking. Thus, trophoblast cells can influence uNK cell function but most likely not uNK cell education. For example, trophoblast cells can influence the MFI through the expression of the trophoblast-specific monomorphic HLA-G, which is key to supporting vascular remodeling, tolerance, and fetal growth (reviewed in [31]).

HLA-C1 allotypes are bound by KIR2DL2/3 and HLA-C2 allotypes by KIR2DL1 and activating KIR2DS1. The immunogenetics of pregnancy should be seen against the background of evolutionary pressure [32]. KIR genes belong to one of two haplotypes, A or B, which are found in all human populations, suggesting that both are important and may be under balanced selection [32]. A working hypothesis suggests that the A haplotypes may be more favorable for immune defense against pathogens, while the B haplotypes may be more favorable for reproduction [32]. Some evidence supporting this idea has been provided. For example, KIR haplotype A is genetically linked to better prognosis in patients with chronic hepatitis C virus [33]. On the other hand, KIR A haplotype is associated with a greater risk for pregnancy complications [34]. For instance, mothers with two KIR A haplotypes and two HLA-C1 allotypes and pregnant with a fetus that has paternally inherited HLA-C2 are more susceptible to developing pre-eclampsia (Figure 2) [35]. Combinations of maternal KIR genotypes and paternal HLA-C genotypes are also associated with birthweight distribution. For example, women with two KIR A haplotypes are more likely to give birth to babies who are small for gestational age [35], while women with at least one copy of KIR B haplotypes are more likely to have babies who are large for gestational age, especially in the presence of paternally derived HLA-C2 [34]. These observations provide an immunogenetic basis for the obstetric dilemma [36].

Despite all this epidemiological evidence, whether the knowledge of KIR and HLA-C genotypes has any clinically relevant predictive power on pregnancy outcomes has not been demonstrated [37]. Nevertheless, a humanized mouse model has directly shown the negative impact of inhibitory KIR–HLA interactions on vascular remodeling and fetal growth [38]. In this elegant model, the interaction between inhibitory KIR2DL1, expressed on maternal uNK cells, and the paternally inherited HLA-C*0501 overly suppressed uNK cells, leading to pathogenic uterine arterial remodeling and changes in uNK cell function [38].

Peripheral NK cells readily recognize and attack cells infected by viruses and cancer cells that have decreased MHC class I expression. Are uNK cells also capable of missing self-recognition, and do they attack fetal trophoblast cells that miss MHC class I expression? What are the effects of inadequate uNK cell education on pregnancy outcomes? Moreover, is uNK cells education imparted by maternal or fetal cells? To address these questions, we recently used a mouse model and have shown that mouse uNK cells do not reject conceptuses lacking the MHC class I expression [39]. We have also shown that the lack of MHC class I expression in the mother causes inadequate uNK cell education, with consequent insufficient vascular remodeling, likely resulting in fetal growth restriction [39]. Furthermore, maternal and not fetal MHC class I molecules educate uNK cells [40]. However, too strong interactions between maternal inhibitory NK cell receptors and MHC class I molecules can increase the risk of fetal growth restriction by suppressing uNK cell function [40].

### 2.4. A New Look: NKG2A and Maternal HLA-E

The majority of human uNK cells (approximately 95%) and half of mouse uNK cells express NKG2A [41]. NKG2A in an inhibitory receptor that binds the non-classical HLA-E, which is also expressed by trophoblast cells [42]. HLA-E binds leader peptides from HLA molecules and needs these for appropriate folding and transport to the cell surface [43,44]. Due to a dimorphism in HLA-B, HLA-E expression and, as a consequence, NKG2A ligation, which leads to NK cell education, differs among the population. Trophoblast cells do not express HLA-B; however, the genetics of HLA-B determines the expression level of HLA-E. About 45% of Caucasians are able to provide functional peptides that result in high HLA-E expression. NKG2A-driven NK cell education results in phenotypically diverse NK cells, which are also functionally more competent in certain conditions [45]. In the rest of the population, low HLA-E expression instead leads to inadequate NKG2A education [45,46]. The HLA-B–HLA-E–NKG2A pathway is probably conserved among and shared by all placental mammals, although the ligand diverges; for example, it is Qa-1 in mice [5,47]. We found that the HLA-B genetic variant responsible for inadequate NKG2A education is associated with a relatively greater risk of pre-eclampsia in a meta-GWAS analysis of >150,000 pregnancies, including >7000 cases of pre-eclampsia (Figure 3). In addition, in a mouse model of NKG2A knock-out dams, we showed that NKG2A is key to educating uNK cell function and for optimal uteroplacental blood flow, fetal growth, and brain development [5].

### 2.5. Transplantation, Tumor Immunology, and the MFI

Historically, our understanding of immunology at the MFI has been formed by the understanding of transplantation immunology. The fetus and its placenta are semi-allogenic since they express paternal genes alongside maternal genes. This has resulted in a strong tradition to view the conceptus as an allograft and pregnancy as an immunological paradox. Therefore, reproductive immunology has been dominated by research on T cells, B cells, and alloimmune responses, while innate immunity cells are the most abundant at the MFI [4].

On the other hand, it may be postulated that by understanding the immunology at the MFI, we may learn new ways to improve transplantation tolerance. In reality, there are fundamental differences between the immunology of transplantation and the immunology at the MFI, which we must recognize to stop making the wrong assumption that mechanisms used at the MFI may be replicated to improve transplantation tolerance and the outcomes of organ transplants. While T cells are attracted to the grafted tissue, only a few T cells are found at the MFI, where innate immune cells like uNK cells and macrophages are most abundant [4]. Moreover, classical MHC class I molecules are expressed by the grafted tissue, whereas trophoblast cells do not express HLA-A or HLA-B, which are ligands for the T-cell receptor. Extravillous trophoblast cells express HLA-C, which binds KIR on uNK cells and T cells—the role of HLA-C restricted T cells at the MFI has been reviewed elsewhere [48], and we do not discuss it here. Extravillous trophoblast cells also express HLA-E, which binds inhibitory NKG2A on uNK cells and potentially on activated T cells, and HLA-G, which binds inhibitory LILRB on uNK cells and macrophages [31]. Thus, the cellular and molecular immunogenetics landscape of the MFI reflects mostly the engagement of innate immunity cells like uNK cells and macrophages.

One specific instance of transplantation that may come close to the immunogenetics at the MFI is hybrid resistance, a laboratory phenomenon discovered in the 1960s that violates the third “law” of transplantation postulated decades earlier. These laws were based on work conducted with inbred mice. The third law postulated that “Transplants from a member of an inbred parental strain to an F1 offspring will succeed but those in the reverse direction will fail” [49]. Indeed, a solid transplant, e.g., kidney, skin, or heart from either parent A or parent B to a hybrid (A×B)F1 offspring would succeed because the alloreactive T cells specific for ‘A’ or ‘B’ parental MHC are deleted in the thymus of the hybrid (A×B)F1 offspring host. However, hematopoietic cells transplanted from either of the two parental inbred mouse strains (A or B) into their irradiated offspring (A×B)F1 hybrid hosts are rejected [50]. After many years, this phenomenon was ascribed to the function of the host NK cells, which would be activated by the recognition of the missing self ‘A’ or ‘B’ MHC on the parental hematopoietic cells [51]. A clinically relevant and analogous setting is haploidentical hematopoietic cell transplantation in leukemia patients [4,52]. In this setting, the donor of choice is half HLA mismatched with the patient. The reason for choosing half HLA mismatched hematopoietic cell transplants is to allow the donor NK cells to attack and destroy the cancer cells in the patient, e.g., to enable a graft versus leukemia reaction. Indeed, because of the half mismatch, some of the inhibitory KIR of the donor’s NK cells will not be engaged by the patient’s HLA class I molecules, and therefore, these donor’s NK cells will not be suppressed and can mediate graft versus leukemia [53]. At the MFI, maternal uNK cells interact with half-HLA mismatched fetal cells, an immunogenetic situation similar to hematopoietic cell transplantation with a half-HLA matched donor. However, the consequence of the mismatch in uNK cell function is not known; it is reasonable to think that a lack of inhibitory uNK cell receptor engagement by fetal trophoblast HLA may be beneficial.

Immunological regulation at the maternal–fetal interface also shows similarities with immune regulation in the tumor microenvironment. Both are dependent on conserved molecular pathways, of which some are also targets of cancer immunotherapy [54]. In highly metastatic lung cancers, the activation of germline and placental genes was identified [55]. Tumor cells also tend to lose classical MHC class I expression. On the other hand, the expression of the non-classical MHC class I HLA-E is a common finding in malignancies and is associated with a poor clinical outcome [55,56]. The HLA-B–HLA-E–NKG2A pathway has a role in the NK cell surveillance of tumor cells [57] and is a target for cancer immunotherapy by inhibitory checkpoint blockade with monoclonal anti-NKG2A antibodies that enhance both NK cell and T cell activity against tumor cells [58].

Although similarities between transplantation, cancer, and the MFI can be drawn, we must remember that reproduction is a mighty selective pressure in the evolution of the species and that the placenta and the immune system have co-evolved for 350 million years. Organ transplantation is a human artifact introduced in the last century or so and, like most cancers (at least those emerging in post-reproductive age), does not contribute to natural selection. Therefore, the immunology of the MFI is unique.

## 3. Conclusions

The immunogenetics at the MFI is unique due to interactions of immune cells with both maternal and fetal cells, with the latter being genetically different from the host. While we appreciate the complexity of the immunogenetic landscape at the MFI and we are starting to look at it in light of new evidence, we lack appropriate assays to fully understand the cellular and molecular mechanisms that are determined by the maternal genetic make-up and modulated by both the unique microenvironment and the fetal genotype. Our incomplete knowledge of MFI physiology impedes a better understanding of the pathological mechanisms leading to pregnancy complications, such as pre-eclampsia and fetal growth restriction. This also prevents us from starting to use effective immunotherapy strategies available in other areas of medicine, such as transplantation and cancer. Once progress in this area is made, one may start to think about intervening with new immunotherapies available to modify the maternal immune system and improve pregnancy outcomes.

## 4. Materials and Methods

### 4.1. Search Methods

The following databases were searched for articles: PubMed, Embase, Web of Science, and Google Scholar. Each database was searched using the following terms: “NK cell”, “fetal maternal interface”, “pregnancy”, “placenta”. The literature search was performed by both authors.

### 4.2. Selection of Papers

Only the literature considered relevant for the discussion of the topic was included.

## Data Availability

No new data were created or analyzed in this study. Data sharing is not applicable to this article.
